# Liver volumetry improves evaluation of treatment response to hepatic artery infusion chemotherapy in uveal melanoma patients with liver metastases

**DOI:** 10.2478/raon-2024-0063

**Published:** 2024-11-28

**Authors:** Sebastian Zensen, Hannah L Steinberg-Vorhoff, Aleksandar Milosevic, Heike Richly, Jens T Siveke, Marcel Opitz, Johannes Haubold, Yan Li, Michael Forsting, Benedikt Michael Schaarschmidt

**Affiliations:** Institute of Diagnostic and Interventional Radiology and Neuroradiology, University Hospital Essen, Germany; Department of Medical Oncology, West German Cancer Center, University of Duisburg-Essen, Essen, Germany; Bridge Institute of Experimental Tumor Therapy, West German Cancer Center, University Medicine Essen, Essen, Germany; Division of Solid Tumor Translational Oncology, German Cancer Research Center (DKFZ) and German Cancer Consortium (DKTK), partner site Essen, Heidelberg, Germany

**Keywords:** uveal melanoma, computed tomography, liver volumetry, staging

## Abstract

**Background:**

In uveal melanoma patients, short-term evaluation of treatment response to hepatic artery infusion chemotherapy (HAIC) using the Response Evaluation Criteria in Solid Tumors (RECIST) 1.1 criteria is challenging due to the diffuse metastatic spread. As liver enlargement can frequently be observed, this study aims to compare RECIST 1.1 and liver volumetry (LV) for the evaluation of HAIC treatment response.

**Patients and methods:**

Treatment response was evaluated in 143 patients (mean age 65.1 ± 10.9 years, 54% female) treated by HAIC by RECIST 1.1 and LV on CT imaging performed before and after HAIC. In LV, different increases in liver volume were evaluated to set an effective threshold to distinguish between stable disease (SD) and progressive disease (PD). Overall survival (OS) was calculated as the time from first HAIC to patient death using Kaplan-Meier test and multivariate analysis was performed for RECIST 1.1 and LV.

**Results:**

In the overall population, median OS (mOS) was 13.5 months (95% CI 11.2–15.8 months). In LV, a threshold of 10% increase in liver volume was suited to identify patients with significantly reduced OS (SD: 103/143 patients, mOS 15.9 months; PD: 40/143 patients, 6.6 months; p < 0.001). Compared to RECIST 1.1, LV was the only significant prognostic factor that was able to identify a decreased OS.

**Conclusions:**

In uveal melanoma patients with liver metastases, LV with a threshold for liver volume increase of 10% was suitable to evaluate treatment response and would be able to be used as a valuable add-on or even alternative to RECIST 1.1.

## Introduction

Uveal melanoma (UM) is the most frequent primary malignancy of the eye and accounts for around 5% of all melanomas.^1,2^ Over the course of the disease, 50% of all patients develop metastases, with the liver being the most common site in 70–90% of cases.^3,4,5^ If liver metastases occur, the prognosis worsens considerately with a 1-year survival rate of about 13% and a median overall survival (mOS) of 2–5 months.^6,7^ Due to their diffuse and infiltrative growth pattern, liver metastases rapidly lead to fatal liver failure. Hence, even in the presence of extrahepatic metastases, aggressive local tumor treatment is key to improve survival.^8^ Due to diffuse metastatic spread, therapies targeting the whole organ such as transarterial chemoembolization (TACE), radioembolization (RE) or hepatic arterial infusion chemotherapy (HAIC) are possible treatment options.^9,10^ Here, especially HAIC plays an important treatment option due to its low rate of side effects that has been shown to prolong progression-free survival with less severe hematologic side effects.^11^

As liver metastases in UM patients often show rapid progression demanding immediate changes in the therapeutic regimen, short-term staging is necessary to evaluate treatment response. However, established tumor response assessment of UM liver metastases using the Response Evaluation Criteria in Solid Tumors (RECIST) 1.1 and its derivatives is challenging. As it is difficult to define a single lesion due to the diffuse liver involvement, a high interobserver measurement variability and thus an inconsistent assessment of response to treatment can be observed.^12,13,14^ However, we observed that considerate liver enlargement occurs in the later stages of the disease. Furthermore, liver volume would be a parameter that could be easily and (potentially automatically) monitored by liver volumetry (LV) over the course of the disease.^15^

To validate if changes in liver volume can also be observed in the earlier stages of the disease, the aim of this study is to compare RECIST 1.1 and LV for the evaluation of treatment response to HAIC in UM patients with liver metastases.

## Patients and methods

### Patient cohort

In this retrospective observational study design, all UM patients who underwent first HAIC for treatment of unresectable UM liver metastases in our department between October 2013 and December 2020 were identified using the Radiology Information System (RIS). Inclusion criteria were: 1) HAIC as only liver directed therapy of liver metastases; 2) no prior surgical therapy of liver metastases; 3) no additional interventions in addition to or during the first HAIC, such as coil embolization of hepatic arteries or use of degradable starch microspheres (DSM); 4) abdominal CT imaging performed no more than 5 days before and at least 5 weeks after first HAIC but before second HAIC. Patients without CT imaging before or after first HAIC were excluded. Ethical approval for this retrospective single-center study was granted by the local ethics committee and the requirement to obtain informed consent was waived (19-8703-BO).

### Hepatic artery infusion chemotherapy

HAIC was performed as described by our research group before via a transfemoral access.^10,16^ Then, a microcatheter was placed either into the proper hepatic artery or selectively into the left and the right hepatic artery and a starting dose of 40 mg melphalan was infused via an automated injector. In our department, HAIC was repeated every 6 to 8 weeks for local tumor control, as this time interval is considered safe and feasible based on pharmacokinetic data from intravenous administration of the chemotherapeutic agent.^17,18^ Before each HAIC, a contrast-enhanced CT scan was performed to assess tumor response and intensify local tumor treatment in case of disease progression.

### Evaluation of treatment response by RECIST 1.1 and liver volumetry

A CT scan was performed one to three days before the first HAIC. The next CT scan was performed 6–8 weeks after the first HAIC without intermediate further local therapy of liver metastases, usually on the day before the second HAIC. All CT scans were acquired in arterial phase of the liver and in venous phase of the whole abdomen. Then, CT images acquired before and after first HAIC were evaluated by LV and RECIST 1.1. using syngo.via (Siemens Healthineers, Erlangen, Germany). LV was performed software-based manually in consensus by two radiologists blinded to outcomes using CT images of the venous phase. RECIST 1.1 evaluation was restricted to the liver. In accordance with RECIST 1.1, the maximum diameter of up to two lesions were analyzed. To correct for perfusion differences, we aimed to assess one lesion in each liver lobe.^19^ To assess the impact of treatment induced changes detectable by RECIST 1.1 and liver volumetry, OS was calculated as the time from first HAIC to patient death. No separate analysis was performed for patients with extrahepatic metastases, as their presence is known not to affect survival.^8^

### Statistics and data analysis

Statistical analysis was performed using GraphPad Prism 5.01 (GraphPad Software, San Diego, USA) and SPSS Statistics 28 (IBM, New York, USA). To determine normal distribution, D’Agostino-Pearson test was applied. Normally distributed data are reported as mean ± standard deviation (SD), non-normally distributed data as median and interquartile range (IQR). Wilcoxon matched pairs test was used to analyze target lesion parameters and liver volumes. Interrater concordance of LV and RECIST 1.1 was assessed by Cohen’s κ-coefficient. Concordance was classified as published by Landis and Koch as no agreement (κ < 0), slight (κ:0.00–0.20), fair (κ:0.21–0.40), moderate (κ:0.41–0.60), substantial (κ:0.61–0.80) or almost perfect (κ:0.81–1.00) agreement.^20^ Overall survival between different groups of liver volume changes, RECIST 1.1 evaluation and combined assessment were compared using Kaplan-Meier curves and log-rank (Mantel-Cox) test. Cox proportional hazards regression model was used to determine hazard ratios (HRs) and the corresponding 95% confidence intervals (CI) of RECIST 1.1 and LV evaluation. A p-value lower than 0.05 was considered statistically significant.

## Results

### Patient cohort characteristics

**FIGURE 1.**
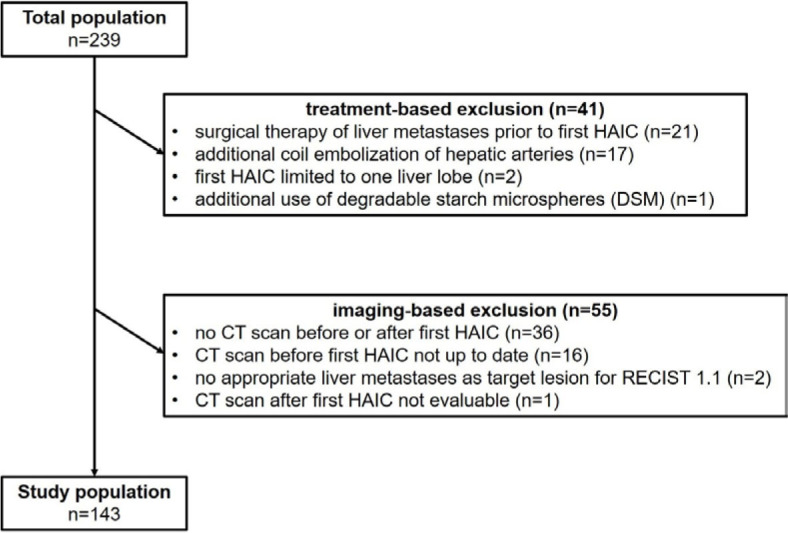
Flowchart of analyzed study population with exclusion criteria. HAIC = hepatic arterial infusion chemotherapy

Between October 2013 and December 2020, 239 patients underwent their first HAIC for the treatment of UM liver metastases, of which 96 patients did not meet the inclusion criteria and were therefore excluded. A total of 143 patients could be included in the analysis (Figure 1).

Treatment-based exclusion criteria were: prior surgical therapy for liver metastases (22%, 21/96), additional coil embolization of hepatic arteries (18%, 17/96), first HAIC limited to one liver lobe (2%, 2/96) and additional use of degradable starch microspheres (DSM) (1%, 1/96). Imaging-based exclusion criteria were: no CT scan before or after first HAIC (38%, 36/96), no current CT scan prior to the intervention (17%, 16/96), no appropriate target lesion for RECIST 1.1 evaluation (2%, 2/96) and CT scan not evaluable due to accompanying liver hematoma (1%, 1/96).

Mean patient age at first HAIC was 65.1 years (SD 10.9, range 28–85) and 54% (77/143) of patients were female. A median number of five HAICs were performed (IQR 3–9, range 1–26). At the time point of data collection (December 2021), a total of 86% (123/143) were deceased, 9% (12/143) were alive and 6% (8/143) were lost to follow-up with a median follow-up time of 1.8 months (IQR 1.6–2.0). Median time period between CT scans before and after first HAIC was 48 days (IQR 44–53). mOS of all patients was 13.5 months (95% CI 11.2–15.8).

### Feasibility of liver volumetry for evaluation of treatment response

**FIGURE 2.**
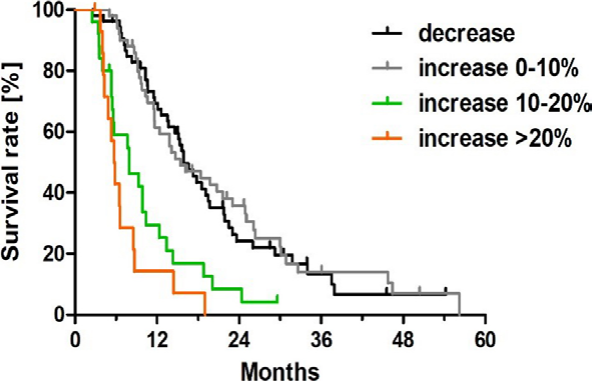
Kaplan-Meier curves of overall survival differentiated by change in liver volume of patients with uveal melanoma with liver metastases after first hepatic artery infusion chemotherapy.

In the entire study population, liver volume before the first HAIC was 1735 ml (IQR 1431–2189 ml, range 889–7116 ml) and after the first HAIC was 1780 ml (IQR 1461–2329 ml, 827–7078 ml, p < 0.0001). The change in liver volume was a median increase of 4% (IQR −2.6% – +11.1%) ranging from a decrease of 20.4% to an increase of 37.6%. First, we performed an explorative data analysis to assess the impact of different changes in liver volume on overall survival using Kaplan-Meier curves (Figure 2).

mOS was comparable for decreasing liver volume (53/143 patients, mOS 15.9 months) and a small increase in liver volume up to 10% (50/143 patients, mOS 15.4 months, p = 0.7852, Figure 2). In contrast, both an increase in liver volume of 10–20% (25/143 patients, mOS 7.9 months, 95% CI 3.6–12.2 months) and more than 20% (15/143 patients, mOS 5.7 months, 95% CI 4.8–6.6 months) were associated with significantly decreased mOS compared to both decreasing or up to 10% increasing liver volume (p < 0.001, Figure 2, Table 1). Accordingly, an increase in liver volume of more than 10% was chosen as the threshold to classify patients as PD by LV, as this was associated with significantly decreased mOS. In contrast, a decrease of liver volume or an increase up to 10% was considered SD. For LV, no patient was evaluated as partial response if a RECIST 1.1 analogue threshold of a 30% decrease in volume was chosen. A complete response for LV is not applicable.

**TABLE 1.** Comparison of median overall survival (mOS) differentiated by different changes in liver volume before and after first hepatic artery infusion chemotherapy

**Liver volume change**			**Log-rank (Mantel-Cox) test**					

			**Increase 0–10%**		**Increase 10–20%**		**Increase > 20%**	

	**N**	**mOS [months]**	**Chi-square**	**p**	**Chi-square**	**p**	**Chi-square**	**p**
Decrease	53	15.9	0.1	0.7852	14.2	0.0002	32.3	< 0.0001
Increase 0–10%	50	15.4			15.8	< 0.0001	33.4	< 0.0001
Increase 10–20%	25	7.9					1.9	0.162
Increase > 20%	15	5.7						

### Treatment response evaluation by RECIST 1.1

**FIGURE 3.**
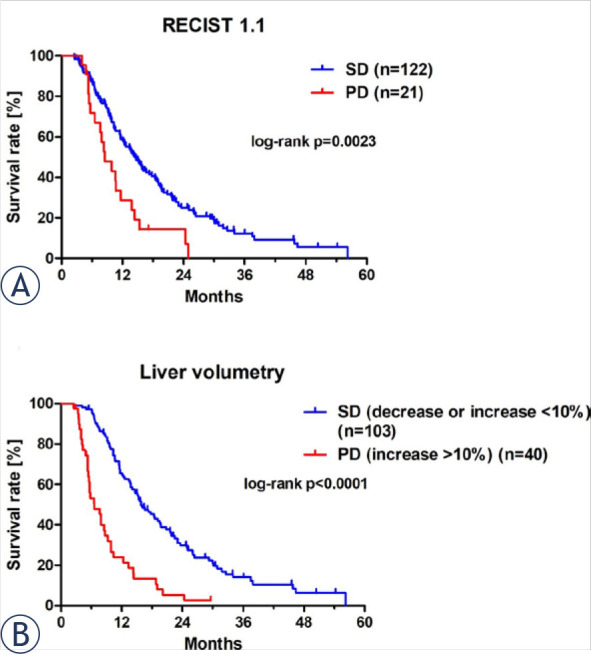
Overall survival of evaluation of treatment response by RECIST 1.1 **(A)** and liver volumetry with a threshold of 10% **(B)** of liver volume increase of uveal melanoma patients with liver metastases treated by hepatic artery infusion chemotherapy. Kaplan-Meier curves show overall survival separately for patients evaluated as stable disease (SD) and progressive disease (PD). In liver volumetry, patients with an increase in liver volume more than 10% were classified as PD and with decrease or increase below 10% as SD. RECIST 1.1 criteria as published.^19^

Measurements of liver target lesions used to evaluate treatment response to HAIC according to RECIST 1.1 are shown in Table 2. In RECIST 1.1, mOS was significantly shorter in patients with PD (8.5 months, 95% CI 5.5–11.5 months, 22/143 patients) than with SD (14.6 months, 95% CI 11.9–17.3 months, 121/143 patients, Chi-square = 9.302, p = 0.0023, Figure 3A). No patient was classified as complete response or partial response according to RECIST 1.1 criteria.

**TABLE 2.** Results of evaluation of treatment response to hepatic artery infusion chemotherapy (HAIC) by RECIST 1.1 and liver volumetry

	**Before first HAIC**	**After first HAIC**	**p-value**
RECIST 1.1^a^			
(Sum of) longest diameter(s) of target lesion(s) [mm]			
Total study cohort (n = 143)	48.6 (IQR 36.3–69.2)	50.8 (IQR 35.3–76.5)	0.0008
SD (n = 122)	47.7 (IQR 36.2–70.5)	48.0 (IQR 34.5–71.1)	0.3485
PD (n = 21)	55.2 (IQR 34.3–68.9)	73.0 (IQR 46.5–85.3)	< 0.0001
Liver volumetry			
Total liver volume [ml]			
Total study cohort (n = 143)	1735 (IQR 1431–2189)	1780 (IQR 1461–2329)	< 0.0001
SD (liver volume decreases or increases up to max. 10%) (n=103)	1678 (IQR 1426–2176)	1714 (IQR 1430–2151)	0.6691
PD (liver volume increases more than 10%) (n=40)	1903 (IQR 1481–2529)	2203 (IQR 1692–2946)	<0.0001

a RECIST 1.1 criteria as published.^19^ Values are given as median and interquartile range (IQR).

SD = stable disease; PD = progressive disease

### Treatment response evaluation by liver volumetry with a threshold of 10% increase in liver volume

Liver volume measurements used to evaluate treatment response to HAIC according to LV with a threshold of 10% increase in liver volume are shown in Table 2. In LV, mOS was significantly shorter in patients with PD (6.6 months, 95% CI 4.4–8.8 months, 40/143 patients) than with SD (15.9 months, 95% CI 12.7–19.1 months, 103/143 patients, Chi-square = 39.28, p < 0.001) (Figure 3B). Initial liver volumes prior to the initial HAIC between patients with PD (1903 ml, IQR 1481–2529 ml) and SD (1678 ml, IQR 1426–2176 ml) were not significantly different (p = 0.2007, Table 2).

Combined treatment response evaluation by RECIST 1.1 and liver volumetry

**FIGURE 4.**
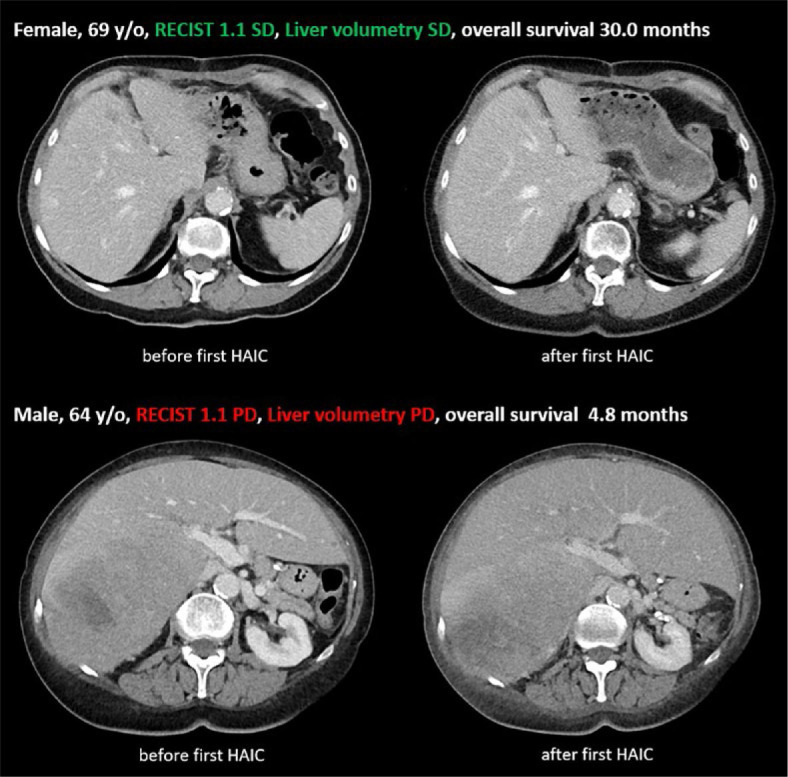
Image examples of CT examinations in portal venous phase before and after first hepatic artery infusion chemotherapy (HAIC) for two patients with evaluations according to RECIST 1.1 and liver volumetry (LV) as stable disease (SD) and progressive disease (PD). Liver metastases are marked with arrows.

Two image examples of concordant evaluations by RECIST 1.1 and LV are shown in Figure 4.

The agreement of LV with RECIST 1.1 was only considered as fair according to the interrater reliability analysis with about a quarter (35/143) of discordant evaluations (κ=0.289, 95% CI: 0.118–0.461, Table 3).

**TABLE 3.** Median overall survival and accordance of treatment response evaluation by RECIST 1.1 and liver volumetry with a threshold of 10% increase in liver volume

**Liver volumetry**			
	SD (Liver volume decreases or increases up to max. 10%)	PD (Liver volume increases more than 10%)	Total
RECIST 1.1 criteria			
SD	16.6 months (n = 95)	6.6 months (n = 27)	14.6 months (n = 122)
PD	12.8 months (n = 8)	7.7 months (n = 13)	8.5 months (n = 21)
Total	15.9 months (n = 103)	6.6 months (n = 40)	12.6 months (n = 143)

Cohen’s κ = 0.289, 95% CI = 0.118–0.461

SD = stable disease; PD = progressive disease

**FIGURE 5.**
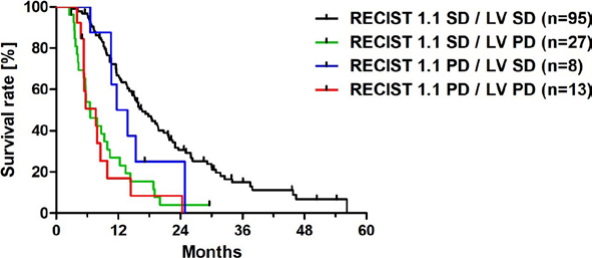
Kaplan-Meier curves for the combined RECIST 1.1 and liver volumetry (LV) evaluation of treatment response with a threshold of 10% increase in liver volume in uveal melanoma patients with liver metastases treated by hepatic artery infusion chemotherapy. PD = progressive disease; SD = stable disease.

Therefore, we further compared the discordant evaluations with both RECIST 1.1 and LV applied in combination. Here, in patients with SD according to RECIST 1.1, mOS was still significantly shorter if changes in LV were > 10% and therefore considered as PD according to LV (RECIST 1.1 SD / LV PD: mOS 6.6 months, 27/143 patients) compared to LV SD (RECIST 1.1 SD / LV SD: mOS 16.6 months, 95/143 patients, Chi-square=28.45, p<0.001) (Table 3, Figure 5).

In contrast, for all cases with changes < 10% in LV, mOS was not significantly different regardless the results of the RECIST 1.1 assessment: (RECIST 1.1 PD / LV SD: mOS 12.8 months, 8/143 patients, RECIST 1.1 SD / LV SD: mOS 16.6 months, 95/143 patients, Chi-square=1.84, p = 0.175, Table 3, Figure 5). Univariate Cox hazard regression analysis indicated that both RECIST 1.1 and LV showed high prognostic value, whereas in the subsequent multivariate analysis only LV remained an independent prognostic factor (Table 4).

**TABLE 4.** Univariate and multivariate Cox proportional hazards regression model of evaluation of treatment response to hepatic artery infusion chemotherapy by RECIST 1.1 and liver volumetry

**Analysis**				**Univariate**			**Multivariate**		
Covariate	Category	n	Median OS (95% CI)	HR (95% CI)		p	HR (95% CI)		p
RECIST 1.1^a^	SD	122	14.6 (11.9–17.3)		Reference			Reference	
	PD	21	8.5 (5.5–11.5)	2.11	1.29–3.45	0.003	1.19	0.92–1.55	0.184
Liver volumetry	SD (liver volume decreases or increases up to max. 10%)	103	15.9 (12.7–19.1)		Reference			Reference	
	PD (liver volume increases more than 10%)	40	6.6 (4.4–8.8)	1.84	1.51–2.26	<0.001	1.77	1.43–2.19	< 0.001

a RECIST 1.1 criteria as published.^19^

HR = hazard ratio; OS = overall survival; PD = progressive disease; SD = stable disease

## Discussion

HAIC is an important and valuable palliative treatment option for liver metastases in patients with UM.^1,18,21^ Short-term assessment of treatment response can be difficult using the commonly used RECIST 1.1 criteria because lesion delineation is challenging due to diffuse organ involvement, leading to increased, reader-dependent measurement variability and inconsistent treatment response evaluation.^12,13,14^ The results of our study can be subsumed in three key points. First, when selecting 10% increase in liver volume as the threshold for PD in LV, more patients with significantly lower OS are identified than by RECIST 1.1. Second, LV and RECIST 1.1 show only fair agreement in the evaluation of treatment response to HAIC. Third, even patients with RECIST 1.1 SD have significantly lower OS when an increase in liver volume of 10% or more is observed in LV.

Tumors that involve the liver often show an asymmetrical and heterogeneous necrosis pattern, which complicates a precise evaluation of treatment response in follow-up imaging.^12^ Therefore, in patients with disseminated liver metastasis, as in UM, measurements of target lesions are often not reliable, making accurate assessment of treatment response difficult.^22^ However, growing liver metastases lead in parallel to an enlargement of the liver volume, which can be easily assessed by LV.^22,23^

Our results show that increases in liver volume of up to 10% are not associated with significantly reduced OS compared to decreasing liver volume. However, a threshold of 10% liver volume increase in LV is well suited to identify patients with significantly reduced OS. Therefore, for the clinical application of LV to evaluate treatment response, we propose a volume increase of 10% as the threshold to distinguish between PD and SD in UM patients with liver metastases. For liver metastases in CRC, LV was also shown to be useful for evaluating treatment response, and the threshold for differentiating between SD and PD was 9.5% liver volume gain, which was very similar to our finding.^22^ Our results show that LV as well as RECIST 1.1 are suitable for evaluation of treatment response but show only moderate interrater reliability with about a quarter of discordant cases. Here, LV can identify more patients than RECIST 1.1 whose life expectancy is significantly decreased. Our data show that even if patients are evaluated as PD by RECIST 1.1, they do not have a significantly decreased OS if their liver volume does not increase by more than 10%. However, if patients are considered SD by RECIST 1.1, but their liver volume increases by more than 10%, their OS is still significantly decreased. These findings are underlined by Cox regression analysis. Here, both LV and RECIST 1.1 have a high prognostic value in assessing treatment response after HAIC. However, after subsequential multivariate analysis, only LV remained an independent prognostic factor. Hence, LV might be a helpful tool to identify non-responders to HAIC that might profit from treatment escalation or potentially other treatment approaches such as RE, radiotherapy or surgery, which are established concepts in other hepatic malignancies apart from hepatocellular carcinomas.^9,24,25,26^ Additionally, when local treatment options are no longer feasible, systemic therapies such as immunotherapy or targeted therapies may offer further treatment possibilities.

Furthermore, liver volumes between SD and PD evaluated patients were not significantly different before the first HAIC, so initial liver volume was not a predictor of significant liver volume change in this study.

Although RECIST 1.1 is the widely used and standardized method for assessing response to treatment in oncologic, LV not only offers additional information but also has methodological advantages compared to RECIST 1.1: LV is a robust method that can be performed as part of the usual CT imaging performed for staging. As the whole organ is assessed, common problems in RECIST 1.1 evaluations leading to inaccurate therapy response evaluation such as varying contrast or poorly delineated lesions due to diffuse organ involvement as well as inter- and intrareader variability can be circumvented by LV.^27,28,29^ In addition, intrareader variability, which is a frequent problem in RECIST 1.1 measurements, might be reduced and thus improve patient response assessment.^30^ Here, especially advancing developments in software and artificial intelligence might transform LV into an automatically acquired datapoint.^31,32,33^ This would allow LV to be easily included as an additional parameter in staging and clinical practice. Despite these promising initial results, these approaches are nevertheless so far experimental and are therefore neither established in clinical routine nor ready for clinical use.

The limitations of our study are its retrospective and single-center study design. Evaluation by LV and RECIST 1.1 was performed by the same radiologist for each of the examinations to avoid interobserver variability. Therefore, these data should be confirmed in prospective studies once automated software solutions for liver volumetry are commercially available. Furthermore, evaluation of treatment response was assessed only after the first HAIC, so follow-up studies should confirm applicability to later time periods in treatment and course of the disease.

In conclusion, in UM patients with liver metastases, LV might be a suitable and in the future robust method to evaluate treatment response by a reliable identification of non-responders to HAIC and a consecutively shortened life expectancy. Hence, it can be used as a valuable add-on or even alternative to RECIST 1.1 to evaluate treatment response in this patient cohort. A threshold for liver volume increase of 10% was effective in distinguishing PD from SD in UM patients with liver metastases.
